# The Evolution of Primary Care Telehealth Disparities During COVID-19: Retrospective Cohort Study

**DOI:** 10.2196/43965

**Published:** 2023-05-17

**Authors:** Rachel D'Amico, Patrick M Schnell, Randi Foraker, J Nwando Olayiwola, Daniel E Jonas, Seuli Bose Brill

**Affiliations:** 1 Department of Internal Medicine The Ohio State University College of Medicine Columbus, OH United States; 2 Division of Biostatistics College of Public Health The Ohio State University Columbus, OH United States; 3 Division of General Medical Sciences Washington University School of Medicine St Louis, MO United States; 4 Humana, Inc Columbus, OH United States

**Keywords:** primary care, health care disparities, COVID-19, office visits, telemedicine, telehealth, health inequality, patient care, telehealth access, health care access

## Abstract

**Background:**

Telehealth has become widely used as a novel way to provide outpatient care during the COVID-19 pandemic, but data about telehealth use in primary care remain limited. Studies in other specialties raise concerns that telehealth may be widening existing health care disparities, requiring further scrutiny of trends in telehealth use.

**Objective:**

Our study aims to further characterize sociodemographic differences in primary care via telehealth compared to in-person office visits before and during the COVID-19 pandemic and determine if these disparities changed throughout 2020.

**Methods:**

We conducted a retrospective cohort study in a large US academic center with 46 primary care practices from April-December 2019 to April-December 2020. Data were subdivided into calendar quarters and compared to determine evolving disparities throughout the year. We queried and compared billed outpatient encounters in General Internal Medicine and Family Medicine via binary logic mixed effects regression model and estimated odds ratios (ORs) with 95% CIs. We used sex, race, and ethnicity of the patient attending each encounter as fixed effects. We analyzed socioeconomic status of patients in the institution’s primary county based on the patient’s residence zip code.

**Results:**

A total of 81,822 encounters in the pre–COVID-19 time frame and 47,994 encounters in the intra–COVID-19 time frame were analyzed; in the intra–COVID-19 time frame, a total of 5322 (11.1%) of encounters were telehealth encounters. Patients living in zip code areas with high utilization rate of supplemental nutrition assistance were less likely to use primary care in the intra–COVID-19 time frame (OR 0.94, 95% CI 0.90-0.98; *P*=.006). Encounters with the following patients were less likely to be via telehealth compared to in-person office visits: patients who self-identified as Asian (OR 0.74, 95% CI 0.63-0.86) and Nepali (OR 0.37, 95% CI 0.19-0.72), patients insured by Medicare (OR 0.77, 95% CI 0.68-0.88), and patients living in zip code areas with high utilization rate of supplemental nutrition assistance (OR 0.84, 95% CI 0.71-0.99). Many of these disparities persisted throughout the year. Although there was no statistically significant difference in telehealth use for patients insured by Medicaid throughout the whole year, subanalysis of quarter 4 found encounters with patients insured by Medicaid were less likely to be via telehealth (OR 0.73, 95% CI 0.55-0.97; *P*=.03).

**Conclusions:**

Telehealth was not used equally by all patients within primary care throughout the first year of the COVID-19 pandemic, specifically by patients who self-identified as Asian and Nepali, insured by Medicare, and living in zip code areas with low socioeconomic status. As the COVID-19 pandemic and telehealth infrastructure change, it is critical we continue to reassess the use of telehealth. Institutions should continue to monitor disparities in telehealth access and advocate for policy changes that may improve equity.

## Introduction

The impact of the SARS-CoV-2 strain of coronavirus (also known as COVID-19) pandemic was felt throughout the world in 2020 and will influence the health care landscape for years to come. The COVID-19 pandemic has highlighted existing health care disparities; even when comorbidities are controlled for, Black patients have 2.7 times increased odds of hospitalization from COVID-19 compared to non-Hispanic White patients [1]. Asian Americans have 2.1 times higher percentage of deaths attributed to COVID-19 compared to non-Hispanic White Americans, with 1 in 7 Asian American deaths in 2020 attributable to COVID-19 [2]. The pandemic has also highlighted the importance of reliable primary care use. Primary care physicians or clinicians manage chronic conditions, like hypertension and diabetes, which are linked with increased mortality secondary to COVID-19 [3]. Primary care physicians are essential in decreasing health care disparities. Increased availability of primary care has been associated with reduced effects of income inequality on self-reported health [4] and all-cause mortality [5]. Despite this, before the COVID-19 pandemic, there were already significant racial and ethnic disparities in primary care access. Urban areas with a high proportion of Black patients were up to 28 times more likely to have limited access to primary care providers [6]. Asian Americans are more likely to be uninsured than non-Hispanic White Americans even after the Affordable Care Act [7]. Given the importance of primary care in patient outcomes and mitigation of health care disparities, it is essential that new models of providing primary care be analyzed critically for equity.

Telehealth is defined by the Centers for Medicare and Medicaid Services as “exchange of medical information from one site to another through electronic communication [8].” At the beginning of the COVID-19 pandemic, telehealth infrastructure and reimbursement developed rapidly. Prior to March 2020, telehealth was reimbursed by Medicare in limited capacities only for patients in designated rural areas. In March 2020, Centers for Medicare and Medicaid Services broadened telehealth access to include all Medicare beneficiaries [8]. The US Department of Health and Human Services also waived restrictions on technology use not compliant with the Health Insurance Portability and Accountability Act to increase options for telehealth platforms [9]. Congress provided US $200 million in April 2020 to help US providers expand telehealth through the COVID-19 Telehealth Program [10]. These policy changes resulted in exponential growth of telehealth. Previous research shows that weekly telehealth visits increased 50-fold for one insurer [11].

Despite the swift increase in telehealth use, studies during the COVID-19 pandemic have demonstrated that Black [12] and Hispanic [13] patients, patients insured by Medicare and Medicaid [14], and those in lower-income areas [12] were less likely to use telehealth within subspecialty care. The data for disparities in adult primary care telehealth use is conflicting but overall concerning for racial inequity [15,16]. In pediatric populations, Black and publicly insured individuals were less likely to use telehealth in primary care [17,18]. However, in patients 65 years of age and older, Black patients used telehealth more frequently than White patients [19]. Little is known about the chronological trends of these disparities. One previous study suggested that the disparities in telehealth lessened throughout 2020 [20].

Our study aimed to further characterize telehealth disparities in adult primary care and to assess how the increase in telehealth use impacted existing health care disparities in primary care, specifically examining differences by race or ethnicity, insurance status, and geographic location in who is using in-person office visits versus telehealth intra–COVID-19. We wanted to determine if any existing disparities were significant only at the beginning of the COVID-19 pandemic related to inequity with initial use or if these disparities continued despite increased clinician and institutional comfort with telehealth use.

## Methods

### Design

We acquired retrospective data from an informational database for the electronic health record of The Ohio State University Wexner Medical Center, which allows researchers to access deidentified clinical data. All billed outpatient encounters in the Division of General Internal Medicine and Department of Family and Community Medicine were examined in 2 time periods: pre–COVID-19 (April-December 2019) and intra–COVID-19 (April-December 2020). These ranges were picked to have comparative time points for the pre- and intra–COVID-19 time frames during the initial peak of the pandemic. Our study aimed to determine how health care disparities may have changed throughout the first year of the COVID-19 pandemic, and data were subdivided into calendar quarters (eg, April-June).

### Participants

Included variables were type of encounter, age, self-identified race, ethnicity, zip code, insurance type, and visit date. Exclusion criteria were ages outside the range of 18-99 years and encounters in departments other than Family Medicine and General Internal Medicine to focus on primary care usage only. Insurance types were separated into Medicaid, Medicare, private, marketplace (including exchange and marketplace policies), worker’s compensation, self-pay, and uninsured.

Our analysis included zip codes for Franklin County, the primary catchment area for patients at The Ohio State University. Franklin County has a population of 1.3 million, of which around 13.5% live below the federal poverty line; 66.8% of the population is White and 23.8% is Black. There are multiple refugee populations located in Franklin County, particularly Nepali (around 23,000 people) and Somali (around 45,000) [12].

### Ethical Considerations

Data were deidentified prior to being given to the research team. The deidentified data request was approved by the Honest Broker Committee (HBOC Study ID #1273), which oversees all clinical data sets within The Ohio State University. Given that the data were deidentified and there were no interventions, the study was designated exempt from institutional review board approval.

### Statistical Analysis

To determine how demographics changed between the pre–COVID-19 and intra–COVID-19 time frames and how they were associated with encounter type (ie, telehealth versus in-person) during the intra–COVID-19 time frame, we applied a binary logistic mixed effects regression model and estimated odds ratios (ORs) and 95% CIs. Time period and encounter type were used as the outcome variables for the respective analyses. Sex, race, and ethnicity of the patient attending each encounter were used as fixed effects. The model was fixed to account for within-office correlation. Because a patient could have multiple encounters, we attempted to fit a mixed effects model in which a random effect for patient was used to account for correlation of encounters by the same patient. However, the mixed effects model indicated no detectable within-patient correlation, so the random effects were dropped from the model.

### Zip Code Analysis

Zip code data and insurance provider were analyzed via separate models. US Census Bureau data for the 48 zip codes within Franklin County was used [21] to determine the effect of geographic socioeconomic status on telehealth use. The percentage of people living below the federal poverty line (FPL) and the percentage of people receiving Supplemental Nutrition Assistance Program (SNAP; eg, “food stamps”) within the zip code were used as indicators of socioeconomic status. Two measurements were used to attempt to fully capture the nuances of socioeconomic disparities. The 10 zip codes with the highest percentage of people living below the FPL or receiving SNAP were classified as “high,” and the 5 zip codes with the lowest percentage of people below FPL and SNAP use were classified as “low,” with all other zip codes in the county categorized as “moderate.” High poverty level was thus identified as >30% of households under FPL, moderate poverty level was considered as 10%-30% of households under FPL, and low poverty level was when <10% of households were under FPL; high SNAP use was >25% of households receiving SNAP, moderate SNAP use was 5%-25% of households receiving SNAP, and low SNAP use was considered as <5% of households receiving SNAP. State and national levels of income were in the moderate categories; 12.6% of the population of Ohio and 11.9% of the US population lived under FPL in 2020 [22], and 13% of the population of Ohio and of the US used SNAP in 2021 [23]. For zip code data, FPL and SNAP were analyzed separately due to multicollinearity (FPL and SNAP categories were highly correlated). Zip codes associated with the university were excluded from analysis to avoid confounding of students artificially lowering income data (those zip codes were classified as a high percentage below FPL and moderate percentage receiving SNAP).

## Results

A total of 81,822 encounters in the pre–COVID-19 time frame and 47,994 encounters in the intra–COVID-19 time frame were analyzed (Table 1). The sample’s racial or ethnic demographics were found to be similar to those of Franklin County, the institution’s primary county [24].

**Table 1:** Comparison of pre–COVID-19 and intra–COVID-19 encounter types.

Characteristics			Pre–COVID-19 (n=81,822), n (%)		Intra–COVID-19 (n=47,994), n (%)		Overall (n=12,9816), n (%)
**Encounter type**							
	Telehealth	9 (0)		5322 (11.1)		5331 (4.1)	
	Office visit	81,813 (100)		42,672 (88.9)		124,485 (95.9)	
**Department**							
	Family medicine	48,821 (59.7)		29,475 (61.4)		78,296 (60.3)	
	General or internal medicine	33,001 (40.3)		18,519 (38.6)		51,520 (39.7)	
**In Franklin County**							
	Yes	61,246 (74.9)		35,271 (73)		96,517 (74.3)	
	No	20,576 (25.1)		12,723 (26.5)		33,299 (25.7)	

### Comparison of Pre–COVID-19 and Intra–COVID-19 Time Frames

There was a substantial decrease in overall primary care encounters during the intra–COVID-19 time frame compared to pre–COVID-19 time frame: between April-December 2019 and April-December 2020, the volume of overall primary care encounters decreased by over 40% (Table 1). Although significant decreases in primary care have been seen nationally, this seems to be a more significant decrease than national trends [25]. There were no significant differences by race or ethnicity between the patients with encounters in the pre–COVID-19 time frame compared to intra–COVID-19 time frame (Tables S1 and S2 in Multimedia Appendix 1). Compared to pre–COVID-19 time frame, there was a higher proportion of patients with marketplace insurance versus private insurance in the intra–COVID-19 time frame (OR 1.11, 95% CI 1.03-1.2; *P*=.006). The proportions of encounters with patients covered by private insurance, Medicare, and Medicaid were similar between pre- and intra–COVID-19 time frames.

Encounters with patients living in Franklin County data were then separately analyzed (Table S1 in Multimedia Appendix 1). Patients living in a zip code area with high utilization rate of SNAP were less likely to use primary care in the intra–COVID-19 time frame compared to those living in a zip code area with low SNAP use (OR 0.94, 95% CI 0.90-0.98; *P*=.006).

### Telehealth Use Disparities

The intra–COVID-19 data (April-December 2020) were then analyzed separately (Table 2). A total of 5322 telehealth and 42,672 office visits were identified. Of note, there were only 9 telehealth encounters in the pre–COVID-19 time frame.

**Table 2:** Racial or ethnic and gender demographics of intra–COVID-19 encounters separated by encounter type.

Characteristics			Telehealth (n=5322), n (%)		Office visit (n=42,672), n (%)		Overall (n=47,994), n (%)
**Sex**							
	Female	3282 (61.7)		24,511 (57.4)		27,793 (57.9)	
	Male	2040 (38.3)		18,160 (42.6)		20,200 (42.1)	
	Unknown	0 (0)		1 (0)		1 (0)	
**Race or ethnicity**							
	White	3704 (69.6)		28,190 (66.1)		31,894 (66.5)	
	African or Black	1056 (19.8)		9312 (21.8)		10,368 (21.6)	
	American Indian	13 (0.2)		78 (0.2)		91 (0.2)	
	Asian	213 (4)		2189 (5.1)		2402 (5)	
	Middle Eastern	33 (0.6)		216 (0.5)		249 (0.5)	
	Multiple	58 (1.1)		403 (0.9)		461 (1)	
	Nepali	15 (0.3)		300 (0.7)		315 (0.7)	
	Somali	12 (0.2)		118 (0.3)		130 (0.3)	
	Pacific Islander	2 (0)		31 (0.1)		33 (0.1)	
	Other	181 (3.4)		1527 (3.6)		1708 (3.6)	
	Unknown	34 (0.6)		305 (0.7)		339 (0.7)	
	Missing	1 (0)		43 (0)		4 (0)	
**Ethnicity**							
	Not Hispanic or Latinx	5146 (96.7)		41,215 (96.6)		46,361 (96.6)	
	Hispanic or Latinx	140 (2.6)		1115 (2.6)		1255 (2.6)	
	Ashkenazi Jew	2 (0)		20 (0)		22 (0)	
	Unknown	34 (0.6)		322 (0.8)		356 (0.7)	

### Racial or Ethnic Disparities

Compared to patients who identified as White, encounters during 2020 with patients of the following racial or ethnic groups were significantly less likely to be via telehealth: Asian (OR 0.74, 95% CI 0.63-0.86; *P*<.001) and Nepali (OR 0.37, 95% CI 0.19-0.72; *P*=.003). In quarterly subanalyses (Table S3 in Multimedia Appendix 1), there was a significant disparity in telehealth use within the same quarter for encounters with Asian patients in quarters 2 and 3 and for Nepali patients in quarters 3 and 4, compared to White patients.

### Insurance Disparities

Compared to private insurance, overall encounters in 2020 with patients covered by Medicare (OR 0.77, 95% CI 0.68-0.88; *P*<.001) were less likely to be via telehealth (Table 3). When analyzed by quarters, encounters with patients insured by Medicare were less likely to be via telehealth in quarters 2 and 3. Although there was no statistically significant difference in telehealth use for patients insured by Medicaid throughout the whole year, subanalysis of quarter 4 found encounters with patients insured by Medicaid were less likely to be via telehealth (OR 0.73, 95% CI 0.55-0.97; *P*=.03).

**Table 3:** Primary insurance coverage of intra–COVID-19 encounters separated by encounter type.

Insurance type	Telehealth (n=5322), n (%)	Office visit (n=42,672), n (%)	Overall (n=47,994), n (%)
Private	2968 (55.8)	21,710 (50.9)	24,678 (51.4)
Marketplace	140 (2.6)	1061 (2.5)	1201 (2.5)
Medicaid	747 (14)	6345 (14.9)	7092 (14.8)
Medicare	1315 (24.7)	12,422 (29.1)	13,737 (28.6)
Other	129 (2.4)	1048 (2.5)	1177 (2.5)
Self-pay	1 (0)	4 (0)	5 (0)
Uninsured	14 (0)	2 (0)	16 (0)
Veterans Affairs	2 (0)	14 (0)	16 (0)
Worker’s compensation	0 (0)	5 (0)	5 (0)
Missing	6 (0)	61 (0.1)	67 (0.1)

### Geographic Disparities

Encounters with patients living in zip code areas with high SNAP use compared to those living in areas with low SNAP use (OR 0.84, 95% CI 0.71 to 0.99; *P*=.04) throughout 2020 were less likely to be via telehealth compared to in-person office visits (Table 4). When comparing quarterly data, there were statistically significant differences in only quarter 4.

**Table 4:** Subanalysis of Franklin County zip code data by encounter type.

Characteristics		Telehealth (n=3937), n (%)	Office visit (n=31,334), n (%)	Overall (n=35,271), n (%)
**FPL^a^**				
	Low	469 (8.8)	3837 (9)	4306 (9)
	Moderate	3042 (57.2)	23,434 (54.9)	26,476 (55.2)
	High	426 (8)	4063 (9.5)	4489 (9.4)
**SNAP use^b^**				
	Low	650 (12.2)	4887 (11.5)	5537 (11.5)
	Moderate	2653 (49.8)	20,761 (48.7)	23,414 (48.8)
	High	634 (11.9)	5686 (13.3)	6320 (13.2)

^a^FPL: federal poverty line; high poverty level was identified as >30% of households under FPL; moderate poverty level was considered as 10%-30% of households under FPL, and low poverty level was considered as <10% of households under FPL.

^b^SNAP: Supplemental Nutrition Assistance Program; high SNAP use: >25% of households receiving SNAP; moderate SNAP use: 5%-25% of households receiving SNAP; and low SNAP use: <5% of households receiving SNAP.

## Discussion

The results of our retrospective cohort study demonstrate that certain patient populations who are more likely to experience health care disparities, including Asian and Nepali patients, those that are insured with Medicare, and patients in areas with high rates of poverty, are not using telehealth at equal rates in primary care settings (Table 5). We found that these disparities persisted throughout 2020. Previous research has shown that disparities in telehealth use in medically underserved areas lessened during 2020 [20]; however, our data contradict this. In addition, patients insured with Medicaid were found to have a new decrease in telehealth use at the end of 2020, suggesting continuing development in disparities that should be explored.

There are several possible explanations for these disparities. Inequitable patient access to telehealth infrastructure is one of the largest concerns. The recent Infrastructure Investment and Jobs Act will allocate US $65 billion for digital-inclusion initiatives, including increasing broadband accessibility [26], in the hopes to curb worsening access disparities. Given the high proportion of refugee populations in Franklin County, including Nepali population, language barriers may make telehealth more difficult to access. As we found disparities in patients insured by Medicare, older patients may lack the education or comfort to adequately use telehealth. Although provider capabilities certainly play a role in telehealth use, one of the strengths of our single center design is that all clinics included in this study had similar telehealth infrastructure and support. With the public health emergency declaration coming to an end in 2022, government officials and insurance payors must decide to what extent telehealth will continue to be reimbursed [27]. If telehealth becomes an increasingly central part of ambulatory care, we may continue to see widening disparities in primary care engagement if telehealth comes at the expense of in-person office visits. Patients must also be included in these conversations to determine what barriers, as well as preferences, affect how they access primary care.

There are some potential opportunities to improve equity within telehealth; quality metric use may improve disparities by allowing providers to offer care options that work best for their patients. Quality metric use was recently shown to decrease racial disparities in postpartum follow-up [28]. Some Medicare plans already incorporate Accountable Care Organizations that have quality metrics [29] that could be modified to be telehealth appropriate. Patient education on electronic portals and telehealth applications is critical to ensuring those with less familiarity with broadband can use telehealth. Interventions as easy as previsit telephone calls have been shown to increase completion of telehealth in underserved populations [30].

Our research does have limitations. As a single-center study, our results may be difficult to generalize to other centers. Additionally, we did not examine the type of care provided during these visits (eg, acute, chronic, and preventative) or if telehealth visits were done by video or telephone, which provides important context. We did not assess patient’s primary language, which may be an additional barrier to telehealth [31]. Our study combined both General Internal Medicine and Family Medicine encounters to develop a comprehensive understanding of primary care telehealth at our institution; however, this may make our data difficult to analyze in the setting of one department.

Nationally, telehealth accounted for 14% of commercially insured ambulatory encounters in 2020 [32], and these rates were maintained into 2021 [33], suggesting that telehealth has entered the mainstream of ambulatory care. The persistent disparities described by our study suggest that ongoing policy discussions about telehealth should focus on health equity to prevent further widening of health disparities in primary care. Our study was also able to examine the changing of telehealth use throughout the year of 2020. By monitoring how the pandemic has continued to evolve, we can determine if changes in telehealth infrastructure are inadvertently affecting specific populations. Continuing to monitor changes in telehealth access is critical to evaluating equity interventions.

**Table 5:** Summary of significant findings that were statistically significant for 2020 and the quarters in which these disparities were found.

Population			Odds ratio (95% CI)		*P* value		Quarters this was statistically significant
**Less likely to access care in intra–COVID-19**							
	Zip code area with high SNAP^a^ use	0.94 (0.9-0.98)		.006		2	
**Less likely to use telehealth**							
	Male gender	0.83 (0.73-0.94)		.003		3 and 4	
	Asian	0.74 (0.63-0.86)		<.001		2 and 3	
	Nepali	0.37 (0.19-0.72)		.003		3 and 4	
	Medicare beneficiaries	0.77 (0.68-0.88)		<.001		2 and 3	
	Zip code area with high SNAP use	0.84 (0.71-0.99)		.04		4	

^a^SNAP: Supplemental Nutrition Assistance Program.

## Appendix

Multimedia Appendix 1 Supplementary material.

## Abbreviations

FPL: federal poverty line
OR: odds ratio
SNAP: Supplemental Nutrition Assistance Program

## Data Availability

The data sets generated and analyzed during this study are available from the corresponding author on reasonable request. Interested parties will be required to complete an institutional Data Use Agreement, and data will be made available via Secure Data transfer.
