# Supplementary material for: The Evolution of Primary Care Telehealth Disparities During COVID-19: Retrospective Cohort Study
Source: J Med Internet Res. 2023 May 17;25:e43965. doi: 10.2196/43965 (PMC10233430; doi:10.2196/43965)
Supplement: Multimedia Appendix 1 [file jmir_v25i1e43965_app1.docx]

**Table S1.** Demographics of all subjects for both pre-COVID-19 and intra-COVID-19 timepoints.

|  | **Pre-COVID-19 (n= 81,822)** | **Intra-COVID-19 (n= 47994)** | **Overall (n= 129816)** |
| --- | --- | --- | --- |
| **Avg age (years)** | 52.1 | 53.2 | 52.5 |
| **Sex** |  |  |  |
| Female | 48722 (59.5%) | 27793 (57.9%) | 76515 (58.9%) |
| Male | 33099 (40.5%) | 20200 (42.1%) | 53299 (41.1%) |
| Unknown | 1 (0.0%) | 1 (0.0%) | 2 (0.0%) |
| **Race/ethnicity** |  |  |  |
| White | 54344 (66.4%) | 31894 (66.5%) | 86238 (66.4%) |
| African/Black | 17921 (21.9%) | 10368 (21.6%) | 28289 (21.8%) |
| American Indian | 176 (0.2%) | 91 (0.2%) | 267 (0.2%) |
| Asian | 4070 (5.0%) | 2402 (5.0%) | 6472 (5.0%) |
| Middle Eastern | 423 (0.5%) | 249 (0.5%) | 672 (0.5%) |
| Multiple | 805 (1.0%) | 461 (1.0%) | 1266 (1.0%) |
| Nepali | 561 (0.7%) | 315 (0.7%) | 876 (0.7%) |
| Somali | 198 (0.2%) | 130 (0.3%) | 328 (0.3%) |
| Pacific Islander | 47 (0.1%) | 33 (0.1%) | 80 (0.1%) |
| Other | 2764 (3.4%) | 1708 (3.6%) | 4472 (3.4%) |
| Unknown | 509 (0.6%) | 339 (0.7%) | 848 (0.7%) |
| Missing | 4 (0.0%) | 4 (0.0%) | 8 (0.0%) |
| **Ethnicity** |  |  |  |
| Not Hispanic/Latinx | 79231 (96.8%) | 46361 (96.6%) | 125592 (96.7%) |
| Hispanic/Latinx | 2012 (2.5%) | 1255 (2.6%) | 3267 (2.5%) |
| Ashkenazi Jew | 23 (0.0%) | 22 (0.0%) | 45 (0.0%) |
| Unknown | 556 (0.7%) | 356 (0.7%) | 912 (0.7%) |
| **Insurance Type** |  |  |  |
| Private | 41492 (50.7%) | 24678 (51.4%) | 66170 (51.0%) |
| Marketplace | 1812 (2.2%) | 1201 (2.5%) | 3013 (2.3%) |
| Medicaid | 12213 (14.9%) | 7092 (14.8%) | 19305 (14.9%) |
| Medicare | 23880 (29.2%) | 13737 (28.6%) | 37617 (29.0%) |
| Other | 2269 (2.8%) | 1177 (2.5%) | 3446 (2.7%) |
| Self-Pay | 23 (0.0%) | 5 (0.0%) | 29 (0.0%) |
| Uninsured | 13 (0.0%) | 16 (0.0%) | 29 (0.0%) |
| VA | 29 (0.0%) | 16 (0.0%) | 45 (0.0%) |
| Worker's comp | 4 (0.0%) | 5 (0.0%) | 9 (0.0%) |
| Missing | 87 (0.1%) | 67 (0.1%) | 154 (0.1%) |
| **% below FPL** |  |  |  |
| Low | 7242 (8.9%) | 4306 (9.0%) | 11548 (8.9%) |
| Moderate | 46273 (56.6%) | 26476 (55.2%) | 72749 (56.0%) |
| High | 7731 (9.4%) | 4489 (9.4%) | 12220 (9.4%) |
| **SNAP usage** |  |  |  |
| Low | 9320 (11.4% | 5537 (11.5%) | 14857 (11.4%) |
| Moderate | 40644 (49.7%) | 23414 (48.8%) | 64058 (49.3%) |
| High | 11282 (13.8%) | 6320 (13.2%) | 17602 (13.6%) |

**Table S2.** Comparison of pre-COVID-19 and intra-COVID-19 encounters by quarter.

|  | **Quarter 2** | | **Quarter 3** | | **Quarter 4** | |
| --- | --- | --- | --- | --- | --- | --- |
|  | **Pre-COVID-19**  **(n=25343)** | **Intra-COVID-19**  **(n= 7018)** | **Pre-COVID-19**  **(n= 26872)** | **Intra-COVID-19**  **(n= 19809)** | **Pre-COVID-19**  **(n= 29607)** | **Intra-COVID-19**  **(n= 21167)** |
| **Encounter Type** |  |  |  |  |  |  |
| Telehealth | 2 (0.0%) | 1959 (27.9%) | 2 (0.0%) | 1760 (8.9%) | 5 (0.0%) | 1603 (7.6%) |
| Office Visit | 25341 (100.0%) | 5059 (72.1%) | 26870 (100.0%) | 18049 (91.1%) | 29602 (100.0%) | 19564 (92.4%) |
| **Department** |  |  |  |  |  |  |
| Family Medicine | 14996 (59.2%) | 4601 (65.6%) | 15951 (59.4%) | 11914 (60.1%) | 17874 (60.4%) | 12960 (61.2%) |
| General/Internal Medicine | 8875 (35.0%) | 1901 (27.1%) | 9157 (34.1%) | 6476 (32.7%) | 9791 (33.1%) | 6685 (31.6%) |
| **Sex** |  |  |  |  |  |  |
| Female | 15231 (60.1%) | 4161 (59.3%) | 16043 (59.7%) | 11574 (58.4%) | 17448 (58.9%) | 12058 (57.0%) |
| Male | 10112 (39.9%) | 2857 (40.7%) | 10828 (40.3%) | 8235 (41.6%) | 12159 (41.1%) | 9108 (43.0%) |
| Unknown | 0 (0%) | 0 (0%) | 1 (0.0%) | 0 (0%) | 0 (0%) | 1 (0.0%) |
| **Race/ethnicity** |  |  |  |  |  |  |
| White | 16711 (65.9%) | 4814 (68.6%) | 17759 (66.1%) | 13134 (66.3%) | 19874 (67.1%) | 13946 (65.9%) |
| African / Black | 5713 (22.5%) | 1434 (20.4%) | 5920 (22.0%) | 4334 (21.9%) | 6288 (21.2%) | 4600 (21.7%) |
| American Indian / Alaska Native | 53 (0.2%) | 12 (0.2%) | 52 (0.2%) | 33 (0.2%) | 71 (0.2%) | 46 (0.2%) |
| Asian | 1229 (4.8%) | 310 (4.4%) | 1356 (5.0%) | 1015 (5.1%) | 1485 (5.0%) | 1077 (5.1%) |
| Middle Eastern | 167 (0.7%) | 42 (0.6%) | 116 (0.4%) | 94 (0.5%) | 140 (0.5%) | 113 (0.5%) |
| Multiple | 250 (1.0%) | 65 (0.9%) | 274 (1.0%) | 183 (0.9%) | 281 (0.9%) | 213 (1.0%) |
| Nepali | 174 (0.7%) | 39 (0.6%) | 187 (0.7%) | 135 (0.7%) | 200 (0.7%) | 141 (0.7%) |
| Native Hawaiian / Pacific Islander | 15 (0.1%) | 7 (0.1%) | 14 (0.1%) | 15 (0.1%) | 18 (0.1%) | 11 (0.1%) |
| Other | 830 (3.3%) | 242 (3.4%) | 936 (3.5%) | 677 (3.4%) | 998 (3.4%) | 789 (3.7%) |
| Unknown | 139 (0.5%) | 36 (0.5%) | 187 (0.7%) | 140 (0.7%) | 183 (0.6%) | 163 (0.8%) |
| Somali | 61 (0.2%) | 16 (0.2%) | 69 (0.3%) | 47 (0.2%) | 68 (0.2%) | 67 (0.3%) |
| Missing | 1 (0.0%) | 1 (0.0%) | 2 (0.0%) | 2 (0.0%) | 1 (0.0%) | 1 (0.0%) |
| **Ethnicity** |  |  |  |  |  |  |
| Not Hispanic/Latinx | 24593 (97.0%) | 6802 (96.9%) | 25990 (96.7%) | 19137 (96.6%) | 28648 (96.8%) | 20422 (96.5%) |
| Ashkenazi Jew | 9 (0.0%) | 1 (0.0%) | 12 (0.0%) | 10 (0.1%) | 2 (0.0%) | 11 (0.1%) |
| Hispanic/Latinx | 586 (2.3%) | 159 (2.3%) | 679 (2.5%) | 523 (2.6%) | 747 (2.5%) | 573 (2.7%) |
| Unknown | 155 (0.6%) | 56 (0.8%) | 191 (0.7%) | 139 (0.7%) | 210 (0.7%) | 161 (0.8%) |
| **Insurance** |  |  |  |  |  |  |
| Private | 12596 (49.7%) | 3484 (49.6%) | 13576 (50.5%) | 10068 (50.8%) | 15320 (51.7%) | 11126 (52.6%) |
| Marketplace | 585 (2.3%) | 174 (2.5%) | 594 (2.2%) | 481 (2.4%) | 633 (2.1%) | 546 (2.6%) |
| Medicaid | 3877 (15.3%) | 1031 (14.7%) | 4089 (15.2%) | 2925 (14.8%) | 4247 (14.3%) | 3136 (14.8%) |
| Medicare | 7576 (29.9%) | 2123 (30.3%) | 7781 (29.0%) | 5844 (29.5%) | 8523 (28.8%) | 5770 (27.3%) |
| Other | 672 (2.7%) | 184 (2.6%) | 774 (2.9%) | 452 (2.3%) | 823 (2.8%) | 541 (2.6%) |
| Self Pay | 5 (0.0%) | 1 (0.0%) | 11 (0.0%) | 2 (0.0%) | 7 (0.0%) | 2 (0.0%) |
| Uninsured | 4 (0.0%) | 3 (0.0%) | 5 (0.0%) | 6 (0.0%) | 4 (0.0%) | 7 (0.0%) |
| VA | 12 (0.0%) | 1 (0.0%) | 9 (0.0%) | 7 (0.0%) | 8 (0.0%) | 8 (0.0%) |
| Workers Comp | 0 (0.0%) | 0 (0.0%) | 2 (0.0%) | 4 (0.0%) | 2 (0.0%) | 1 (0.0%) |
| Missing | 16 (0.1%) | 17 (0.2%) | 31 (0.1%) | 20 (0.1%) | 40 (0.1%) | 30 (0.1%) |

**Table S3.** Comparison of telehealth and office visit encounters during the intra-COVID-19 timeperiod by quarter.

|  | **Quarter 2** | | **Quarter 3** | | **Quarter 4** | |
| --- | --- | --- | --- | --- | --- | --- |
|  | **Telehealth**  **(n= 1959)** | **Office visit**  **(n= 5059)** | **Telehealth**  **(n= 1760)** | **Office visit**  **(n= 18049)** | **Telehealth**  **(n= 1603)** | **Office visit**  **(n= 19564)** |
| **Department** |  |  |  |  |  |  |
| Family Medicine | 1013 (51.7%) | 3588 (70.9%) | 1002 (56.9%) | 10912 (60.5%) | 901 (56.2%) | 12059 (61.6%) |
| General/Internal Medicine | 769 (39.3%) | 1132 (22.4%) | 664 (37.7%) | 5812 (32.2%) | 625 (39.0%) | 6060 (31.0%) |
| **Sex** |  |  |  |  |  |  |
| Female | 1173 (59.9%) | 2988 (59.1%) | 1092 (62.0%) | 10482 (58.1%) | 1017 (63.4%) | 11041 (56.4%) |
| Male | 786 (40.1%) | 2071 (40.9%) | 668 (38.0%) | 7567 (41.9%) | 586 (36.6%) | 8522 (43.6%) |
| Unknown | 0 (0%) | 0 (0%) | 0 (0%) | 0 (0%) | 0 (0%) | 1 (0.0%) |
| **Race/ethnicity** |  |  |  |  |  |  |
| White | 1396 (71.3%) | 3418 (67.6%) | 1204 (68.4%) | 11930 (66.1%) | 1104 (68.9%) | 12842 (65.6%) |
| African / Black | 374 (19.1%) | 1060 (21.0%) | 377 (21.4%) | 3957 (21.9%) | 305 (19.0%) | 4295 (22.0%) |
| American Indian / Alaska Native | 5 (0.3%) | 7 (0.1%) | 3 (0.2%) | 30 (0.2%) | 5 (0.3%) | 41 (0.2%) |
| Asian | 64 (3.3%) | 246 (4.9%) | 72 (4.1%) | 943 (5.2%) | 77 (4.8%) | 1000 (5.1%) |
| Middle Eastern | 12 (0.6%) | 30 (0.6%) | 10 (0.6%) | 84 (0.5%) | 11 (0.7%) | 102 (0.5%) |
| Multiple | 23 (1.2%) | 42 (0.8%) | 20 (1.1%) | 163 (0.9%) | 15 (0.9%) | 198 (1.0%) |
| Nepali | 7 (0.4%) | 32 (0.6%) | 4 (0.2%) | 131 (0.7%) | 4 (0.2%) | 137 (0.7%) |
| Native Hawaiian / Pacific Islander | 0 (0%) | 7 (0.1%) | 1 (0.1%) | 14 (0.1%) | 1 (0.1%) | 10 (0.1%) |
| Other | 63 (3.2%) | 179 (3.5%) | 50 (2.8%) | 627 (3.5%) | 68 (4.2%) | 721 (3.7%) |
| Unknown | 10 (0.5%) | 26 (0.5%) | 13 (0.7%) | 127 (0.7%) | 11 (0.7%) | 152 (0.8%) |
| Somali | 4 (0.2%) | 12 (0.2%) | 6 (0.3%) | 41 (0.2%) | 2 (0.1%) | 65 (0.3%) |
| Missing | 1 (0.1%) | 0 (0%) | 0 (0%) | 2 (0.0%) | 0 (0%) | 1 (0.0%) |
| **Ethnicity** |  |  |  |  |  |  |
| Not Hispanic/Latinx | 1900 (97.0%) | 4902 (96.9%) | 1699 (96.5%) | 17438 (96.6%) | 1547 (96.5%) | 18875 (96.5%) |
| Ashkenazi Jew | 0 (0%) | 1 (0.0%) | 1 (0.1%) | 9 (0.0%) | 1 (0.1%) | 10 (0.1%) |
| Hispanic/Latinx | 47 (2.4%) | 112 (2.2%) | 49 (2.8%) | 474 (2.6%) | 44 (2.7%) | 529 (2.7%) |
| Unknown | 12 (0.6%) | 44 (0.9%) | 11 (0.6%) | 128 (0.7%) | 11 (0.7%) | 150 (0.8%) |
| **Insurance** |  |  |  |  |  |  |
| Private | 1040 (53.1%) | 2444 (48.3%) | 985 (56.0%) | 9083 (50.3%) | 943 (58.8%) | 10183 (52.0%) |
| Marketplace | 45 (2.3%) | 129 (2.5%) | 43 (2.4%) | 438 (2.4%) | 52 (3.2%) | 494 (2.5%) |
| Medicaid | 296 (15.1%) | 735 (14.5%) | 252 (14.3%) | 2673 (14.8%) | 199 (12.4%) | 2937 (15.0%) |
| Medicare | 515 (26.3%) | 1608 (31.8%) | 433 (24.6%) | 5411 (30.0%) | 367 (22.9%) | 5403 (27.6%) |
| Other | 58 (3.0%) | 126 (2.5%) | 38 (2.2%) | 414 (2.3%) | 33 (2.1%) | 508 (2.6%) |
| Self Pay | 0 (0%) | 1 (0.0%) | 1 (0.1%) | 1 (0.0%) | 0 (0%) | 2 (0.0%) |
| Uninsured | 3 (0.2%) | 0 (0%) | 5 (0.3%) | 1 (0.0%) | 6 (0.4%) | 1 (0.0%) |
| VA | 0 (0%) | 1 (0.0%) | 1 (0.1%) | 6 (0.0%) | 1 (0.1%) | 7 (0.0%) |
| Workers Comp | 0 (0%) | 0 (0%) | 0 (0%) | 4 (0.0%) | 0 (0%) | 1 (0.0%) |
| Missing | 2 (0.1%) | 15 (0.3%) | 2 (0.1%) | 18 (0.1%) | 2 (0.1%) | 28 (0.1%) |
